# Lymphoma presenting as preauricular tumor in unilateral parotid gland agenesis: a case report and review of literature

**DOI:** 10.1186/s13256-024-04553-9

**Published:** 2024-05-03

**Authors:** Aris I. Giotakis, Christos Masaoutis, Alexander Delides, Evangelos I. Giotakis

**Affiliations:** 1 First Department of Otorhinolaryngology Head and Neck Surgery, Metropolitan General, Mesogeion 264, 15562 Athens, Greece; 2 Department of Pathology, Metropolitan General, Mesogeion 264, 15562 Athens, Greece; 3 2nd Department of Otolaryngology, Attikon University Hospital, National and Kapodistrian University of Athens-Faculty of Medicine, Rimini 1, 12462 Athens, Greece; 4 First Department of Otorhinolaryngology, Hippocration Hospital, Medical University of Athens, National and Kapodistrian University of Athens, Vas. Sofias 114, 11527 Athens, Greece

**Keywords:** Parotid neoplasms, Small lymphocytic lymphoma, Congenital abnormalities, Sialendoscopy, Case report

## Abstract

**Background:**

Parotid gland agenesis is a rare, congenital, usually asymptomatic disorder. Until now, only 24 cases with unilateral, incidentally found, parotid gland agenesis have been described. Here, we present the first reported case of an ipsilateral preauricular neoplasm in a patient with unilateral parotid gland agenesis. During surgery, the position of the greater auricular- and facial nerves was documented. Furthermore, we performed the first sialendoscopy for this rare disorder to assess the number of duct branches, which might be indicative of the abundance of parotid tissue. Moreover, we looked for sialendoscopic characteristic features that could aid in identifying these patients in the ambulatory setting.

**Case presentation:**

A 50-year-old Greek man presented with a painless, slowly enlarging mass in the right parotid space. Magnetic resonance imaging revealed a complete absence of the right parotid gland without accessory parotid tissue. The right parotid gland was replaced by fatty tissue and the radiologist suggested a benign parotid tumor. Fine needle aspiration was indicative of a reactive lymph node. Sialendoscopy revealed only two branches within the right parotid duct. Surgical resection was performed through a conventional lateral parotidectomy. This revealed typical anatomic position of the greater auricular- and facial nerves despite the parotid tissue agenesis. Histopathology revealed a small lymphocytic lymphoma.

**Conclusions:**

Surgeons should feel confident to resect tumors of the parotid space in patients with parotid gland agenesis. Reduced branching observed during sialendoscopy might indicate parotid gland agenesis. Physicians should be even more cautious than usual with the watch and wait strategy in patients with tumors of parotid gland agenesis, since the probability of a tumor being a benign salivary gland tumor might be lower than usual.

## Background

Agenesis of the parotid gland (PGA) is a rare entity and can be bilateral or unilateral, symptomatic or asymptomatic, total or partial, and with or without presence of papilla of Stensen’s duct [1, 2]. Current data do not associate unilateral PGA with significant pathologies [1, 2]. A pleomorphic adenoma has been described in the accessory parotid gland located in the ipsilateral buccal space of two cases with unilateral PGA [3, 4].

Here, we report the first case of a patient with a neoplasm located directly in the ipsilateral parotid space of unilateral PGA. Furthermore, we describe the spatial relationship of the tumor with the position of the greater auricular- and facial nerves, since there is a paucity of data that describe the position of these nerves in the absence of parotid tissue. Moreover, we report the value of diagnostic sialendoscopy in parotid gland agenesis. Informed consent was signed by the patient. The authors assert that all procedures contributing to this work comply with the ethical standards of the relevant national and institutional guidelines on human experimentation (Ethical Committee of Metropolitan General 358/18072022) and with the Helsinki Declaration of 1975, as revised in 2008.

## Case presentation

In May 2022, a 50-year-old self-employed, married Greek man, working as a psychiatrist, presented with a 3-month history of a painless, slowly enlarging mass in the right parotid space. Allergies, medical conditions, daily medication, previous surgeries, recent trauma or infection were not reported. The patient did not mention any specific dental history except regular dental cleaning. The patient denied smoking and alcohol consumption.

Physical examination revealed a 2 cm × 1 cm firm, mobile, and nontender mass located anteriorly and caudally of the right ear lobe. Both papillae of Stensen’s duct were present. The outer ear was free of wax and inflammation. We noted no signs of inflammation or perforation of the tympanic membrane, and the middle ear was filled with air. We did not observe any septal deviation in the nose and the nasal mucosa was neither inflamed nor swollen. A fiberoptic examination revealed a normal nasopharynx, and the larynx as well as the hypopharynx were free of pathologies. The oral mucosa was normal. We noted a tonsillar grade II hypertrophy, without pus. The rest of the oropharyngeal mucosa was normal. Palpation of the neck as well as of the left parotid area revealed no further pathologies. The patient reported no neurologic condition. Therefore, we did not refer the patient to a neurologist for neurologic examination. Pulse was 65 bpm, blood pressure was 112/75, and temperature was 36.6 °C.

**Fig. 1:**
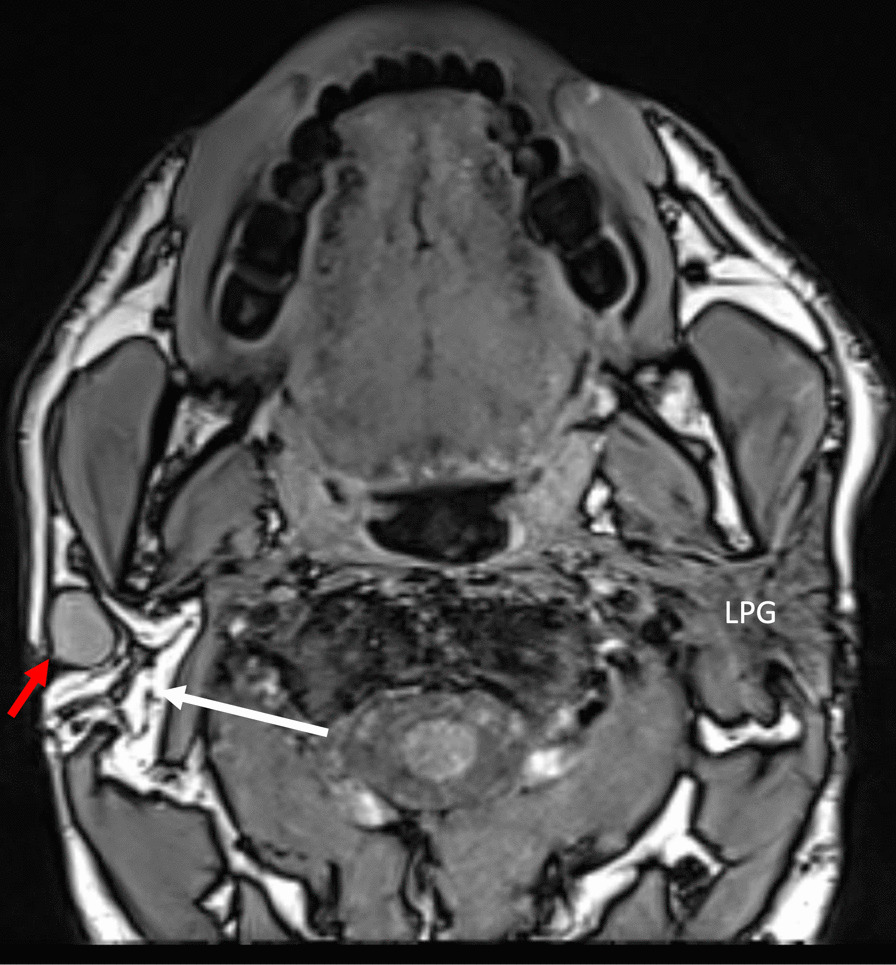
Magnetic resonance imaging of the neck with gadolinium (T1 sequence) revealed a normal left parotid gland (LPG). In the right parotid space, no parotid gland was noted. Instead, a rather homogeneous well-defined mass (indicated by the small red arrow) was surrounded by fatty tissue (indicated by the longer white arrow)

Magnetic resonance imaging (MRI) of the neck with gadolinium revealed a complete absence of the right parotid gland without accessory parotid tissue (Fig. 1). The left parotid gland was normal. The right parotid gland was replaced by fatty tissue, suggestive of PGA. A well-defined, 1.7 cm × 1.2 cm × 1 cm, rather homogeneous, solid mass in the right parotid space surrounded by fatty tissue was noted (Fig. 1). The radiologist suggested a benign parotid tumor. Fine needle aspiration guided by ultrasound was indicative of a reactive lymph node.

Surgical resection was planned under general anesthesia. Preoperative blood tests, including complete blood count and liver and renal function, electrocardiography, and chest X-ray were normal, except for a slightly higher percentage of lymphocytes in the peripheral blood (45%; normal range: 25–40%). Urinalysis, serology as well as microbiologic examinations were not performed systematically in the preoperative check-up.

The number of branches within the duct might indicate the degree of parotid aplasia/hypoplasia. This could provide additional information about the abundance of parotid tissue, which could be useful during surgery. Therefore, a sialendoscopy was performed. Reduced saliva secretion through the right papilla during dorsoventral massage of the right parotid space compared with the left side was noted. During sialendoscopy, the duct’s color, diameter, and elasticity noted by its reaction to normal saline as well as its course by the masseter muscle appeared normal. As far as the endoscope could reach, two branches were noted. Transillumination was intense.

**Fig. 2:**
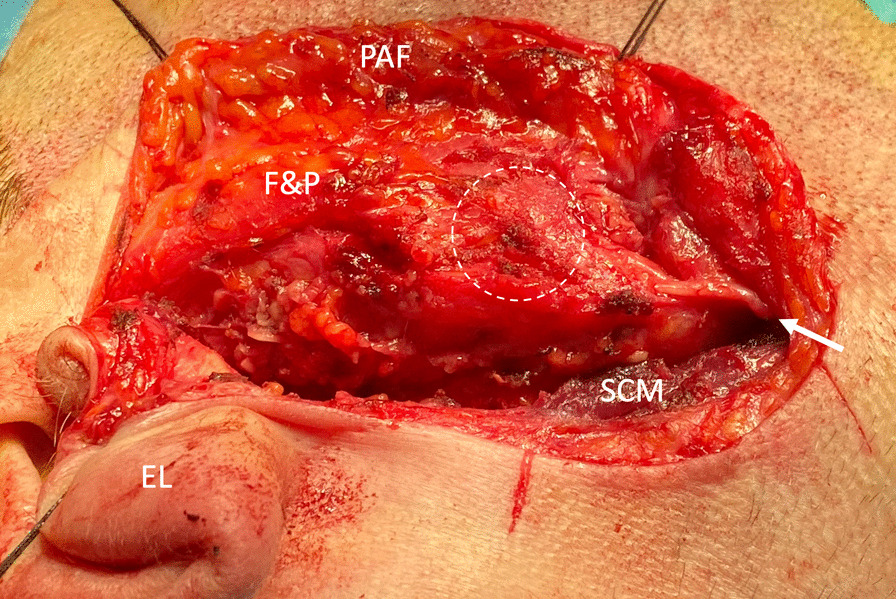
Right side. The ear lobe and the preauricular flap were retracted by fixing sutures. The tumor is indicated by the dashed circle. The white arrow pointed at the greater auricular nerve EL, ear lobe; PAF, preauricular flap; F&P, partially fatty and partially parotid tissue; SCM, sternocleidomastoid muscle

**Fig. 3:**
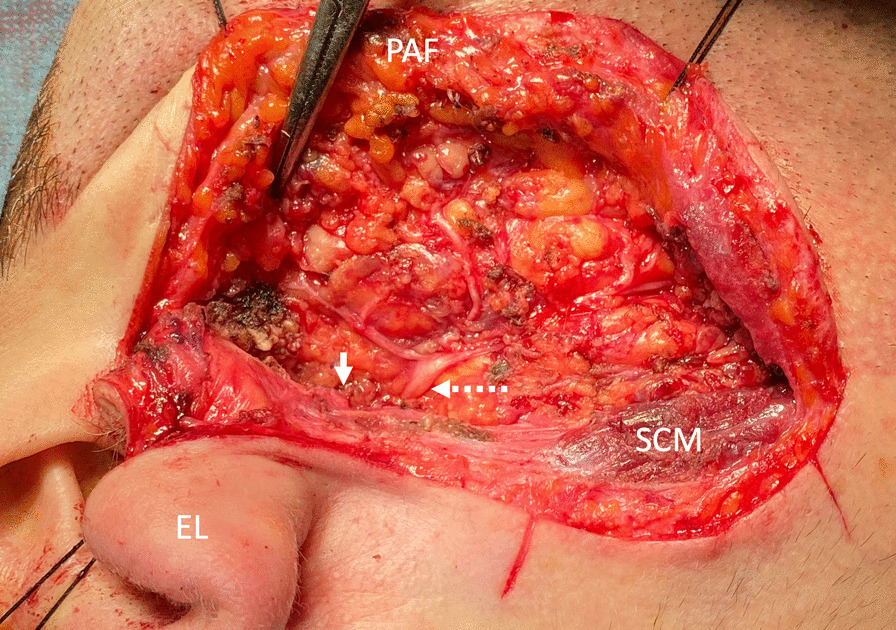
Right side. The ear lobe and the preauricular flap were retracted by fixing sutures. After resection of the tumor and surrounding tissue located laterally to the facial nerve. The small white arrow indicates the pointer. The white dashed longer arrow indicates the facial nerve EL, ear lobe; PAF, preauricular flap; SCM, sternocleidomastoid muscle

Tumor resection was planned through a modified Blair incision. This was carried out in an ordinary fashion as if it were a lateral parotidectomy. In the parotid space beneath the preauricular flap, a mixture of fatty- and parotid tissue instead of the usual parotid tissue was noted (Fig. 2). The greater auricular nerve followed its typical course (Fig. 2). The facial nerve was found in its typical place, medially and caudally to the pointer (Fig. 3). The facial nerve was dissected free from its lateral partially fatty- and partially parotid tissue (Fig. 3). The 4.5 cm × 4.5 cm specimen with the tumor was sent for histopathologic evaluation.

No complications were noted postoperatively. The drain was removed on the second postoperative day. Subsequently, the patient was discharged. After surgery, the patient intravenously received 1 g of paracetamol at 16:00 and 24:00 h, as well as 40 mg of parexocib at 21:00 h. He also intravenously received 2.2 g of amoxicillin/clavulanic acid at 15:30 and 23:30 h. On the first postoperative day, he intravenously received 1 g of paracetamol every 8 h as well as 2.2 g of amoxicillin/clavulanic acid every 8 h. On the second postoperative day, prior to drain removal he intravenously received 2.2 g of amoxicillin/clavulanic acid. After discharge, he orally received 1 g of amoxicillin/clavulanic acid every 8 h for 5 days. He was advised to follow an acidic-free and sour-free nutrition for 2 weeks.

**Fig. 4:**
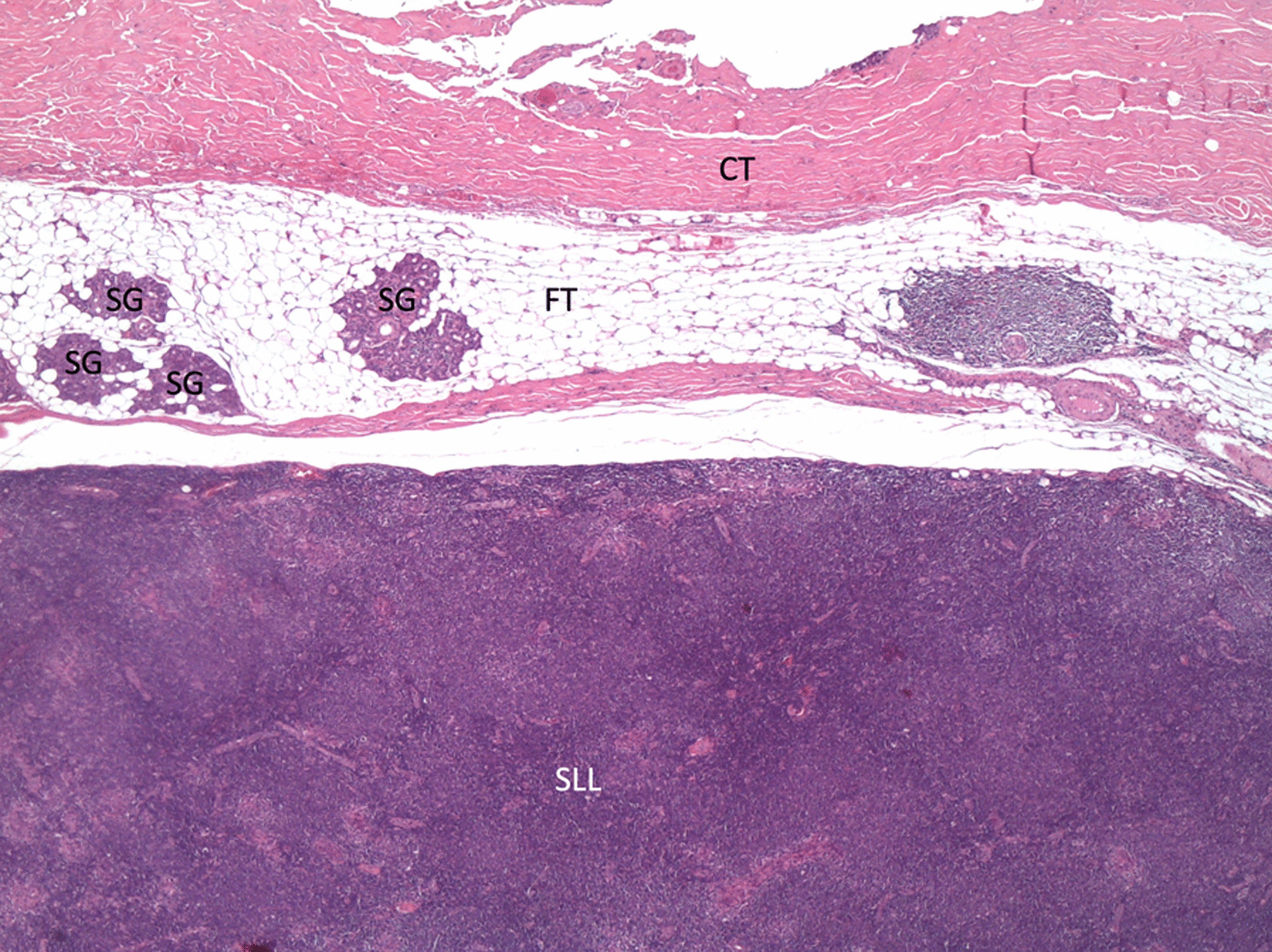
Hematoxylin and eosin staining, × 20. A lymph node with disrupted architecture can be seen in the lower half. Atrophic / hypoplastic salivary gland parenchyma within the perinodal fat is noted on the upper left quadrant SG, salivary gland; FT, perinodal fat; CT, connective tissue; SLL: small lymphocytic lymphoma

The histopathology examination revealed a lymph node with partially disrupted architecture on account of a small lymphocytic proliferation with interfollicular/perifollicular and vaguely nodular distribution, staining positive for CD20, CD5, CD23, and LEF1 on immunohistochemistry. Therefore, the diagnosis of chronic lymphocytic leukemia/small lymphocytic lymphoma (CLL/SLL) was reached. The lymph node was surrounded by fatty tissue with small islets of parotid tissue (Fig. 4).

The patient was informed about the findings and revealed that his parents were also diagnosed with CLL in the past. The patient was referred to the hematology department for treatment. A positron-emission-tomography/computed-tomography scan with 18 fludeoxyglucose as a radiotracer revealed no further pathology in the body. The patient received no treatment and has remained under regular examination by the hematologists since then.

## Discussion and conclusions

To date, this is the first case that described the presence of a neoplasm in the ipsilateral parotid space of unilateral PGA. Major salivary gland agenesis was first described by Gruber in 1885 [5]. Unilateral PGA typically remains asymptomatic. Therefore, its actual incidence is difficult to assess. Here, a data search in the PubMed database was performed by using all possible combinations of the following keywords: “parotid,” “gland,” “agenesis,” “aplasia,” and “unilateral.” Only human studies in English were evaluated. Overall, 12 out of 39 results were considered relevant.

In their case report and literature review, Teymoortash and Hoch summarized the manifestations of unilateral PGA [1]. These may include symptoms of the contralateral parotid gland, such as sialosis [6], hypertrophy, and pleomorphic adenoma [7]. Furthermore, symptoms of the ipsilateral accessory parotid tissue in the buccal space have been reported, such as pleomorphic adenoma [4] and recurrent inflammation [8]. Moreover, multiple pathologies of the head, such as contralateral cheek lipoma [1] and metastases to the region [9], have been the causes for revealing unilateral PGA. Additionally, physicians should not neglect that PGA might be associated with other systemic congenital abnormalities, such as Down or Klinefelter syndrome and lacrimo-auriculo-dento-digital syndrome 1 [2].

Interesting epidemiological characteristics include the even distribution between men and women, the wide spectrum of age at time of diagnosis (50 days to 75-years old with an average of 35 years), and the two times more frequent predominance of the right over the left side [2]. Diagnosis is usually confirmed by radiologic imaging. MRI provides crucial information about PGA [1]. Alternatively, computed tomography (CT) can be also used [3]. Despite the accuracy of ultrasound in assessment of parotid tumors [10], no data are available for its utility in PGA.

In this case report, FNA was suggestive of a lymph node. Owing to the MRI findings, surgical resection for diagnosis and potential treatment was planned. Before surgery, a sialendoscopy was performed. The aim was to assess the number of branches, which might have been indicative of the degree of parotid aplasia/hypoplasia. Reduced branching was noted. The other duct features, e.g., color, diameter, and elasticity, were rather normal. Therefore, the only duct sialendoscopic finding that might indicate toward PGA was the reduced branching. Comparison of the sialendoscopic features of both parotid ducts would be interesting. However, this was not performed owing to absent pathology. These findings suggested that more data are needed before sialendoscopy is characterized as a valuable or not valuable procedure for detection of PGA in the ambulatory setting.

Obviously, the discussion about the value of diagnostic sialendoscopy in PGA, which is an asymptomatic disease, is rather academic with questionable clinical utility. On the contrary, the description of the surgical excision steps of this preauricular tumor in unilateral PGA is of high clinical importance. Several questions may emerge when MRI reveals such tumors and their surrounding environment. Unprepared physicians might question the quality of the MRI, and patient’s sincerity or even mental status, i.e., if the patient has had a parotidectomy. Head and neck surgeons should be familiar with salivary gland agenesis. Even then, the most suitable surgical approach is an important question. Surgeons might be troubled by the position of the greater auricular nerve and the facial nerve, e.g., superficial or not, as well as the best approach for tumor excision, e.g., enucleation or lateral parotidectomy.

Considering the disease and paucity of similar data, wariness was essential during surgical resection. In similar cases, surgeons should expect a dominance of fatty tissue over parotid tissue (Fig. 2). Surgeons should feel confident to perform an ordinary lateral parotidectomy through a modified Blair or modified facelift incision. The greater auricular nerve (Fig. 2) and facial nerve (Fig. 3) should be expected in their typical courses. Since there have been no other reports where tumors were surgically resected from the parotid space of unilateral PGA, comparison with other studies is not feasible.

Owing the aplasia/hypoplasia of the salivary gland in PGA, the probability of a tumor being a salivary gland tumor might be lower than usual. To date, there has not been a report that described the presence of a salivary tumor in the ipsilateral parotid space of unilateral PGA [1, 2]. Therefore, physicians should be even more cautious than usual in recommending the watch and wait strategy in PGA tumors.

Concluding, the current case report suggested that surgeons should not hesitate to surgically treat tumors located in the parotid space of PGA, since the crucial anatomic features here remain unchanged. MRI or CT are crucial as verifiable and not examiner dependent methods. The only duct sialendoscopic finding that might indicate PGA was reduced branching. More data are needed for sialendoscopy to be characterized as valuable or not valuable diagnostic procedure for PGA. Future surgeons should feel encouraged to gather more data by surgery and diagnostic sialendoscopy.

## Data Availability

The data used and/or analyzed during the current study are available from the corresponding author on reasonable request.

## Abbreviations

PGA: Parotid gland agenesis
MRI: Magnetic resonance imaging
CLL/SLL: Chronic lymphocytic leukemia/small lymphocytic lymphoma
CT: Computed tomography
